# From flowers to function: Structural and biomedical exploration of iron oxide nanoparticles synthesized from *Rhododendron arboreum* extract

**DOI:** 10.1016/j.jtumed.2025.09.005

**Published:** 2025-10-16

**Authors:** Iram Saba, Vivek K. Dhiman, Susmitha Kalaichelvan, Rajasekaran Subbarayan, Ankush Chauhan, Ritesh Verma, Khalid Mujasam Batoo, Saif Hameed, Ahmed A. Ibrahim

**Affiliations:** a Amity Institute of Biotechnology, Department of Biotechnology, Amity University Haryana, Gurugram (Manesar), Haryana - India; b Centre for Herbal Pharmacology and Environmental Sustainability, Department of Herbal Pharmacology and Environmental Sustainability, Chettinad Hospital and Research Institute, Chettinad Academy of Research and Education, Tamil Nadu, India; c Centre for Advanced Biotherapeutics and Regenerative Medicine, Department of Advanced Biotherapeutics and Regenerative Medicine, Chettinad Hospital and Research Institute, Chettinad Academy of Research and Education, Tamil Nadu, India; d Faculty of Allied Health Sciences, Department of Allied Health Sciences, Chettinad Hospital and Research Institute, Chettinad Academy of Research and Education, Tamil Nadu, India; e Department of Physics, Graphic Era (Deemed to be University), Dehradun-248002, India; f College of Science, King Saud University, P.O. Box 2455, Riyadh-11451, Saudi Arabia; g Department of Physics and Astronomy, King Saud University, Riyadh-11451, Saudi Arabia

**Keywords:** التخليقالأخضر, جسيماتأكسيدالحديدالنانوية, رودودندرونأرابوريم, مضاد, مضادللبكتيريا, α-Fe_2_O_3_nanoparticles, Antibacterial, Anticancer, Green synthesis, *Rhododendron arboreum*

## Abstract

**Objectives:**

This study was aimed at developing a green, ecologically friendly method for synthesizing iron oxide nanoparticles (α-Fe_2_O_3_ NPs) by using *Rhododendron arboreum* flower extract, as well as evaluating their potential biomedical applications.

**Method:**

X-ray diffraction, scanning electron microscopy, energy dispersive X-ray spectroscopy, ultraviolet-visible spectroscopy, and Fourier-transform infrared spectroscopy were used to characterize the α-Fe_2_O_3_ NPs synthesized from the *Rhododendron arboreum* flower extract.

**Results:**

Comprehensive characterization of the synthesized α-Fe_2_O_3_ NPs revealed a hydrodynamic diameter of 274.7 nm and a zeta potential of −18.6 mV. X-ray diffraction analysis confirmed the formation of crystalline α-Fe_2_O_3_ with an average crystallite size of 32.03 nm. The nanoparticles exhibited potent antibacterial activity against *Bacillus subtilis, Staphylococcus aureus, Pseudomonas aeruginosa,* and *Salmonella typhi*, with lower minimum inhibitory concentration and minimum bactericidal concentration values than the flower extract, thus indicating enhanced antimicrobial efficacy. Anti-inflammatory potential, assessed with egg albumin denaturation assays, indicated a significant decrease in inflammation. Furthermore, cytotoxicity evaluations in MCF-7 breast cancer cell lines indicated an IC_50_ value of 22 μg/mL, suggesting promise for cancer therapy applications.

**Conclusion:**

These findings highlight the successful green synthesis of α-Fe_2_O_3_ NPs with potential biomedical applications in antibacterial, anti-inflammatory, and anticancer treatments.

## الملخص

**أهداف البحث:**

تهدف هذه الدراسة لتوضيح كيفية تخليق جسيمات أكسيد الحديد النانوية بطريقة صديقة للبيئة باستخدام مستخلص زهرة شجرة رودودندرون كعامل اختزال طبيعي.

**طرق البحث:**

تم استخدام حيود الأشعة السينية، والمسح المجهري الإلكتروني، والتحليل الطيفي للأشعة السينية المشتتة للطاقة، والتحليل الطيفي فوق البنفسجي المرئي، والتحليل الطيفي للأشعة تحت الحمراء لتوصيف جسيمات أكسيد الحديد النانوية المصنعة من مستخلص زهرة شجرة رودودندرون.

**النتائج:**

كشف التوصيف الشامل لجسيمات أكسيد الحديد النانوية التي تم تصنيعها عن قطر هيدروديناميكي يبلغ 274.7 نانومتر وجهد كهروحركي يبلغ −18.6 مللي فولت. أكد تحليل حيود الأشعة السينية تكوين بلورات أكسيد الحديد النانوية البلوري بحجم بلورات متوسط يبلغ 32.03 نانومتر. أظهرت الجسيمات النانوية نشاطًا مضادًا قويًا ضد البكتيريا العصوية الرقيقة، وبكتيريا المكورة العنقودية، وبكتيريا الزائفة الزنجارية، وبكتيريا السالمونيلا التيفية حيث أظهرت قيم تركيز الحد الأدنى المثبط وتركيز الحد الأدنى القاتل للبكتيريا نسبة أقل مقارنةً بمستخلص الزهور، مما يشير إلى الفعالية المحسنة لجسيمات أكسيد الحديد النانوية كمضادات للميكروبات. كما تم تقييم الإمكانات المضادة للالتهابات باستخدام فحص تمسخ زلال البيض، مما كشف عن انخفاض كبير في الالتهاب. علاوة على ذلك، أظهرت تقييمات السمية الخلوية على خطوط خلايا سرطان الثدي أن قيمة نصف التركيز التثبيطي الأقصى تبلغ 22 ميكروغرام/مل، مما يشير إلى تطبيقات واعدة في علاج السرطان.

**الاستنتاجات:**

تسلط هذه النتائج الضوء على التخليق الأخضر الناجح لجسيمات أكسيد الحديد النانوية مع التطبيقات الطبية الحيوية المحتملة في العلاجات المضادة للبكتيريا والالتهابات ومضادات السرطان.

## Introduction

Nanotechnology, a multidisciplinary field, has witnessed remarkable advancements enabling wide-ranging applications across domains including agriculture, manufacturing, engineering, medicine, and therapeutics.^1^ At the core of nanotechnology lie nanoparticles (NPs), clusters of atoms or molecules with dimensions of 1–100 nm.^2^ These nanomaterials exhibit unique physicochemical properties that distinguish them from their bulk counterparts, primarily due to their high surface area-to-volume ratio. Attributes such as charge distribution, zeta potential, particle size, morphology, crystallinity, and thermal stability contribute to their enhanced functionality in various scientific and industrial applications.^3^

Healthcare is among the most promising nanotechnology applications, particularly targeted drug delivery, disease diagnostics, and therapeutic interventions.^4^^,^^5^ However, conventional methods for synthesizing NPs often involve hazardous chemicals, which pose substantial health and environmental risks. According to Mary et al.,^6^ NPs synthesized by using toxic reagents may retain residual contaminants on their surfaces, thus potentially causing adverse biological effects. Consequently, an increasing shift toward environmentally sustainable synthesis approaches, particularly those inspired by natural processes, is occurring.^7^^,^^8^

Among various ecologically friendly approaches, plant-mediated green synthesis of NPs has gained substantial attention for its cost-effectiveness, sustainability, and biocompatibility.^9^ Using plant extracts as reducing and stabilizing agents in NP synthesis offers an efficient alternative to conventional chemical methods. Various plant-derived biomolecules, including polyphenols, terpenoids, phenolic acids, and alkaloids, are critical in facilitating metal ion reduction and NP stabilization. Extracts obtained from flowers, roots, leaves, and stems have been successfully used in the biosynthesis of metal and metal oxide NPs.^10^^,^^11^

Green synthesis using plant extracts presents several advantages, such as eliminating the need for complex purification steps, decreasing energy consumption, and using readily available natural resources. Additionally, this approach enables precise control of NP morphology, size distribution, and stability, and therefore is an attractive option for biomedical, environmental, and industrial applications. By harnessing the potential of plant-based synthesis, researchers can develop safe, efficient, and scalable methods for producing NPs while mitigating the ecological footprint associated with conventional synthetic techniques. These methods are preferred for green biosynthesis, because they stabilize dispersible NPs, and facilitate the oxidation or reduction of metal ions during synthesis. The presence of biomolecules, including polyphenols, terpenoids, phenolic acids, and alkaloids, in plant extracts, is the reason for the reduction–oxidation phenomenon.^12^, ^13^, ^14^ Plant extract-mediated synthesis provides cost benefits over conventional synthesis methods by eliminating the need for additional purification steps and using readily available plant-based raw materials.^15^ Furthermore, the ability to effectively control NP development and dispersal makes this synthesis method safe and environmentally sustainable.^16^

Bacterial infections are caused by pathogenic bacteria invading the body and disrupting normal physiological processes. These infections range from mild to life-threatening, and affect various systems, including the respiratory tract (e.g., *Streptococcus pneumoniae*), gastrointestinal system (e.g., *Escherichia coli* and *Salmonella typhi*), and the bloodstream (e.g., *Staphylococcus aureus*). Nanoparticles exhibit remarkable antimicrobial activities and have been combined with antibiotics and herbal formulations to treat these infections.^17^, ^18^, ^19^ Iron oxide (Fe_2_O_3_) exhibits excellent conductivity, owing to its wide energy band gap, and therefore is highly suitable for applications in electrical, optoelectronic, and electrochemical devices.^20^ Fe_2_O_3_ NPs frequently display distinct properties from a large bulk of the material, because of their higher surface area-to-volume ratio.^21^ Studies on the bioactivity of Fe_2_O_3_ NPs have revealed their significant antibacterial effects, particularly against Gram-positive bacteria.^22^^,^^23^

*Rhododendron arboreum*, a flowering plant with vibrant red blossoms, belongs to the Ericaceae family. The Indigenous people of North India extensively use this plant for both culinary and traditional medicinal reasons. The blossoms are used to produce jellies, local beer, and jams in the mountainous regions of Himachal Pradesh.^24^ In traditional medicine, the blossoms are used for managing dysentery and diarrhea, whereas the dried blossoms are consumed to treat bleeding dysentery.^25^ *Rhododendron arboreum* has been shown to have diuretic, choleretic, anti-irritable bowel syndrome, and astringent properties, and to be beneficial in treating chronic diarrhea.^26^ A wide range of plant products have been effectively documented as capping agents for NP stabilization in the literature.^27^ Nevertheless, few studies in the literature have focused on metal-based NPs derived from *Rhododendron arboreum*. This plant is distributed globally but is concentrated primarily in China, India, Malaysia, and Nepal.^28^ The *Rhododendron arboreum* flowers, in comparison to other plant parts, contain a diverse array of phytochemicals, such as flavonoids, tannins, and phenolic compounds, which have been recognized for their reducing and stabilizing properties. These phytochemicals assist in decreased concentration of iron ions throughout the synthesis process and inhibit the agglomeration of NPs, thus enhancing product consistency and stability.^29^ The structural characteristics of the biogenic Fe_2_O_3_ NPs were examined with Fourier-transform infrared spectroscopy (FTIR), X-ray diffraction (XRD), scanning electron microscopy (SEM), dynamic light scattering (DLS), and zeta potential methods. The bioactivity of the NPs was evaluated by testing their antibacterial and anti-inflammatory properties. This study describes FeO NPs synthesized from extracts from *Rhododendron arboretum* flowers, which contain a unique combination of reducing/stabilizing agents (e.g., flavonoids such as quercetin and phenolic acids), thus enabling faster NP formation at room temperature than other extracts requiring heating or prolonged reaction times.^30^ In this study, we aimed to develop a green, ecologically friendly method for synthesizing iron oxide (α-Fe_2_O_3_) NPs by using *Rhododendron arboreum* flower extract, as well as to evaluate their potential biomedical applications.

## Materials and Methods

### Chemicals and reagents

Ferric nitrate nonahydrate (Fe(NO_3_)_3_·9H_2_O) was purchased from Sisco Research Laboratories Pvt. Ltd. (SRL) – India; sodium hydroxide (NaOH) pellets were purchased from HIMEDIA®; methanol (CH_3_OH) was purchased from Amichem Research Lab; Lurai Bertani broth was purchased from SRL; and Mueller-Hinton agar was purchased from HIMEDIA®.

### Preparation of *Rhododendron arboreum* methanolic extract

The flowers of *Rhododendron arboreum* were collected from the Solan area in Himachal Pradesh, India. The plant was identified at Nauni University in Solan, Himachal Pradesh, India, with accession no. 0698. The flowers of *Rhododendron arboreum* underwent a shade-drying process followed by pulverization with an electric grinder. The resultant 10 g dried flower powder was dispersed in 100 mL methanol and placed in a shaking incubator at 40 °C for 48 h. Furthermore, the plant supernatant was filtered with filter paper and dried in a hot air oven at 40 °C for 48 h. The dried extract was stored at 4 °C until subsequent use.

### Synthesis of Fe_2_O_3_ NPs

**Figure 1:**
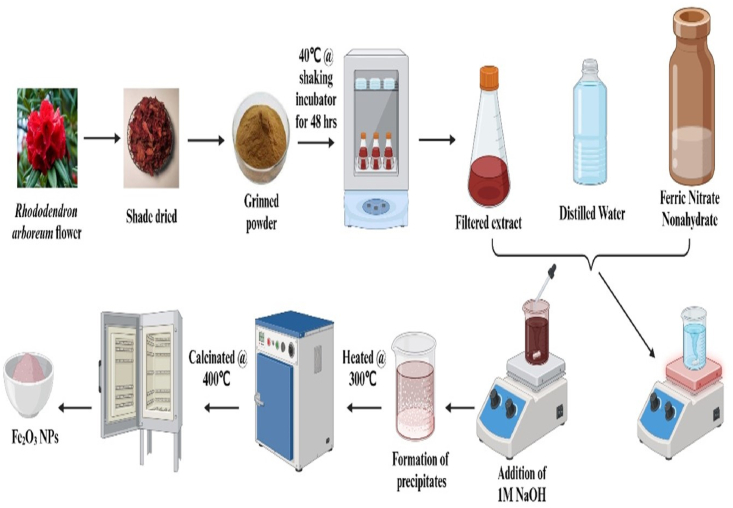
Schematic illustration of the synthesis of Fe_2_O_3_ NPs.

For synthesis of iron oxide NPs, an aqueous solution was prepared by dissolving 10 g ferric nitrate nonahydrate in 100 mL distilled water. To this solution, 10 mL *Rhododendron arboreum* flower extract (1 mg/mL concentration) was added under continuous stirring for 2 h. The pH was adjusted to 8 by addition of a 1M aqueous solution of NaOH. Brownish precipitates formed, and the solution was stirred continuously for 2 h. The precipitates were washed with distilled water and ethanol, under centrifuged, and the resultant precipitates dried at 300 °C in a hot air oven. The dried powder was ground with a mortar and pestle and subjected to calcination in a muffle furnace at 400 °C for 4 h (Figure 1).

### Material characterization

The hydrodynamic diameter and surface charge analysis were determined with DLS (Malvern Zetasizer). The detection of unique chemical bonds was performed through FTIR spectroscopy, with Bruker Alpha equipment. In addition, XRD patterns were studied to confirm the phase composition of the synthesized Fe_2_O_3_ NPs. The structure and morphology of the Fe_2_O_3_ NPs were analyzed with SEM.

### Antibacterial activity

#### Bacterial strains

Gram-positive bacteria, including *B. subtilis*, *S. aureus* (ATCC 25923), and Gram-negative bacteria such as *Pseudomonas aeruginosa* (ATCC 27853) and *Salmonella* Typhi (ATCC 6539), were obtained from HiMedia Laboratories Pvt. Ltd., Mumbai, India. These bacterial strains were cultured in nutrient broth (HiMedia, Mumbai, India) at 37 °C for 24 h under constant agitation at 200 rpm.

#### Minimum inhibitory concentration assays and microbial bactericidal concentration (MBC) evaluation

The minimum inhibitory concentration (MIC) of an antimicrobial agent is the lowest concentration required to prevent the growth of an organism in culture tubes or micro-dilution setups. The MIC was used to compare the antibacterial activity of rhododendron flower extract and green-synthesized Fe_2_O_3_ NPs. A sterile 96-well plate was prepared with 100 μL Mueller-Hinton broth added to each well designated for bacterial pathogens. Test samples (98 μL) were added and then diluted 1:2. Control wells (triplicates) were maintained in separate vertical columns. The first well in each column contained 98 μL test sample, and 100 μL was subsequently transferred to the next well to achieve serial dilution. Finally, a 2 μL volume of bacterial pathogens was added to each well. The plates were incubated at 35 °C for 24 h to allow for bacterial growth. After incubation, the MIC values were determined as the lowest concentration at which no visible bacterial growth was observed. Minimum bactericidal concentration (MBC) assays were conducted to further evaluate bacterial survival. Agar plates were inoculated with samples from wells showing no growth and incubated to confirm bacterial death or inhibition. Results were recorded after incubation to determine the bactericidal efficacy.

#### Agar well diffusion assays

The antibacterial effectiveness of Fe_2_O_3_ NPs was assessed with the agar well diffusion method, as previously described.^27^ Cotton swabs were used, and the bacterial strains were streaked onto Mueller-Hinton agar. An aseptic pipette tip was used to perforate the agar. Each well was filled with 10 μL plant extract, 10 μL NPs, 10 μL ampicillin (positive control), and 10 μL DMSO (negative control). The Petri dishes were incubated at 37 °C for 24 h, after which the zone of inhibition was observed.

### Protein denaturation activity

#### Egg albumin denaturation assays

The effectiveness of the NPs in preventing the denaturation of egg albumin protein was investigated with a modified version of the method described by Velidandi et al.^28^ A standard inhibitory test involved adding 112 μL PBS buffer, 8 μL egg white solution, and various concentrations (6.75, 12.5, 25, 50, or 100 μL) of NPs and plant extract from a 100 mg/mL stock solution to a 96 well plate. The plate was incubated for 15 min at 37 °C, then exposed to a temperature of 80 °C for 5 min in a water bath and allowed to cool. The samples’ absorbance at a wavelength of 660 nm was determined. A reaction tube containing aspirin served as the positive control.

(1) $$\text{Protein}\;\text{Denaturation}\;\text{Inhibition}\;\%=\left(\frac{Ac-As}{Ac}\right)*100$$

where *Ac* is the absorbance of the control, and *As* is the absorbance of the sample.

### MTT cell viability assays of Fe_2_O_3_NPs

The tetrazolium salt 3-[4, 5-dimethyl-2-thiazolyl]-2,5-diphenyl-2H-tetrazolium bromide (MTT)^29^ was used to assess the cytocompatibility of Fe_2_O_3_NPs in the MCF-7 cell line. The cells were cultured in high glucose DMEM, and 10,000 cells/mL were plated in 96 well plates and treated with various concentrations of Fe_2_O_3_NPs (control, 1.56, 3.125, 6.2, 12.5, 25, 50, and 100 μg/mL) for 24 h. The medium was replaced, and 10 μL MTT (5 mg/mL stock solution) was added to each well. The plates were then incubated for 4 h to allow the formation of formazan crystals within the cells. After incubation, the crystals were dissolved by addition of 150 μL dimethyl sulfoxide (DMSO) to each well, and the optical density (OD) was measured at a 50 nm wavelength. The percentage cell viability was calculated with the following formula: % viability = (mean OD sample/mean OD blank) × 100.

### Alamar blue assays for the assessment of anticancer-effects of Fe_2_O_3_NPs

Alamar blue assays were used to study the cell proliferation efficiency of the MCF-7 cell line in the presence of Fe_2_O_3_NPs. In 96-well plates, MCF-7 cells were seeded at 0.25 × 10^6^ cells/mL and placed in a CO_2_ incubator. After incubation, the cells were provided with fresh medium with various concentrations of Fe_2_O_3_NPs (0, 1, 5, 10, or 25 μg/mL). Alamar blue was directly added to the culture medium at a final concentration of 10 % and incubated for 10–15 min. The OD was measured at an excitation wavelength of 560 nm and an emission wavelength of 590 nm, according to the manufacturer's instructions (G-Biosciences, MO, USA Cat #786–921).^30^

### AO/PI staining

Cells were seeded at a density of 1 × 10^6^ cells/well in a six-well plate. After 24 h of incubation, the medium was replaced with fresh medium containing Fe_2_O_3_NPs (1, 5, 10, or 25 μg/mL). After 24 h incubation, the cells were washed with 1 × PBS and treated with a mixture of 10 μg/mL acridine orange and 10 μg/mL propidium iodide (dissolved in PBS). The cells in the six-well plate were washed with 1 × PBS. Fluorescence images were captured with a Nikon Epi-fluorescence inverted microscope, and data were recorded.^31^

### Statistical analysis

All experimental data were collected in triplicate and are presented as mean ± standard deviation (SD), unless otherwise stated. Statistical analyses were conducted in GraphPad Prism software version 9.0 (GraphPad Software, San Diego, CA, USA). One-way analysis of variance (ANOVA) was used to determine statistically significant differences among experimental groups. Subsequently, Tukey's multiple comparisons post hoc test was conducted to identify pairwise differences between the control and treated groups. The compared groups included untreated controls, *Rhododendron arboreum* flower extract-treated cells, Fe_2_O_3_ NP-treated cells, and cells subjected to combination treatments, as applicable. This approach was used across all biological assays, including antibacterial (zone of inhibition, MIC, and MBC), anti-inflammatory (egg albumin denaturation), and cytotoxicity (MTT assay) analyses.

A p value < 0.05 was considered statistically significant. Results with p < 0.01, p < 0.001, and p < 0.0001 were noted as increasingly significant and are marked in the figures and legends. Non-significant differences are reported as “ns.” All statistical comparisons and significance levels are clearly indicated in the corresponding figure legends and result descriptions.

## Results

### Characterization

#### FTIR analysis

**Figure 2:**
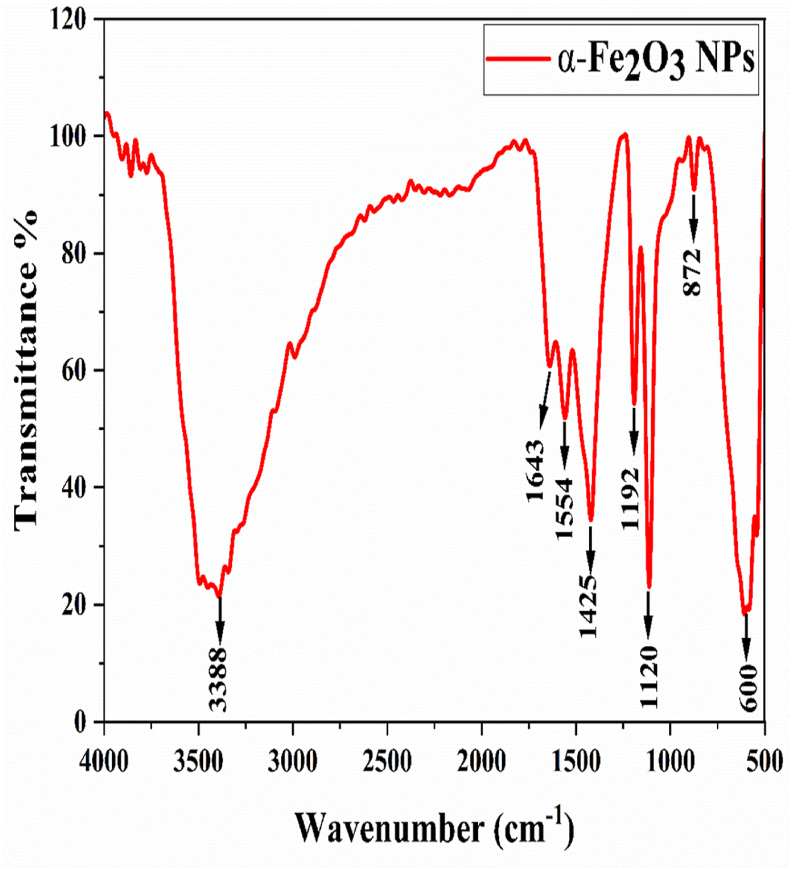
FTIR spectrum of α-Fe_2_O_3_ nanoparticles.

The FTIR spectrum of α-Fe_2_O_3_ NPs produced from *Rhododendron arboreum* flowers displayed clear absorption peaks, thereby providing essential information regarding the chemical composition and bonding properties of the NPs (Figure 2). For instance, the peak at 3388 cm^−1^, representing O–H stretching vibrations, indicates the presence of alcohol.^32^ The peak observed at 1638 cm^−1^, representing C=C stretching, indicates the presence of polyphenols.^33^ The peak observed at 1554 cm^−1^, represents N–O stretching, indicates the presence of nitroalkanes.^34^ The peak observed at 1425 cm^−1^, representing O–H bending, indicates the presence of carboxylic acid.^35^ The peak observed at 1192 cm^−1^, representing C–O stretching, indicates the presence of the ester class.^36^ The peak observed at 1120 cm^−1^, representing C–O stretching, indicates the presence of secondary alcohol.^37^ The peaks observed at 872 cm^−1^ and 600 cm^−1^ might have indicate the presence of Fe–O bonds.^32^^,^^33^^,^^38^

#### DLS and zeta analysis

**Figure 3:**
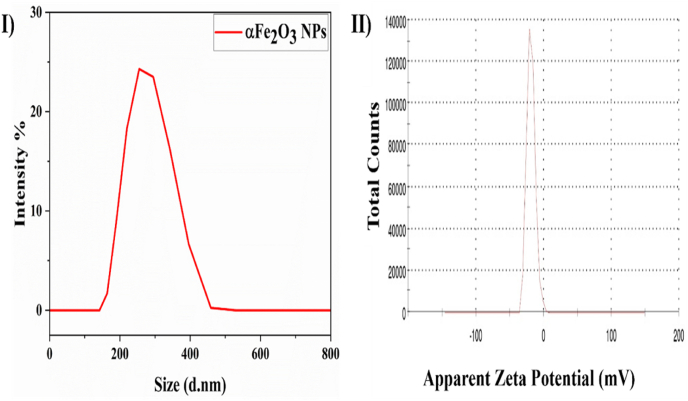
(I) DLS Analysis of α-Fe_2_O_3_ nanoparticles and (II) zeta analysis of α-Fe_2_O_3_ nanoparticles.

Figure 3 (I and II) indicates a hydrodynamic diameter of Fe_2_O_3_ NPs of 274.7 nm and a zeta potential of −18.6 mV, thus providing critical insights into the physical characteristics and stability of Fe_2_O_3_ NPs obtained from *Rhododendron arboreum* flowers. Figure 3 (I) shows a hydrodynamic diameter of Fe_2_O_3_ NPs of 274.7 nm, indicating a relatively large particle size. Figure 3 (II) depicts the electric charge on the surfaces of NPs in a colloidal dispersion. The negative zeta potential indicates that the NPs possessed a net negative surface charge, thus implying possible electrostatic repulsion among particles and resistance to aggregation.

#### XRD analysis

**Figure 4:**
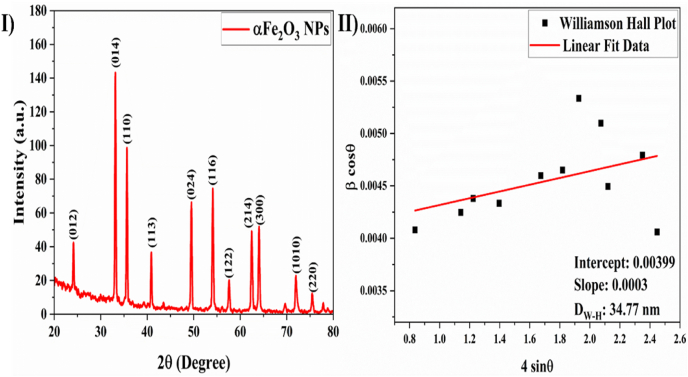
(I) XRD pattern and (II) W–H plot of α-Fe_2_O_3_ nanoparticles.

The XRD pattern depicted in Figure 4 (I) confirmed the successful synthesis of Fe_2_O_3_ NPs via the green synthesis method. The observation of a crystalline phase with a well-defined diffraction profile indicated high crystallinity and phase purity. The diffraction peaks at 24.07, 33.2, 35.7, 40.7, 49.5, 54.1, 57.6, 62.4, 63.9, 72.02, and 75.4 belonged to the (012), (014), (110), (113), (024), (116), (122), (214), (300), (1010), and (220) (hkl) planes of the Fe_2_O_3_ crystal, respectively, in good agreement with hematite phase JCPDS file no. 84–0311. The presence of sharp crystalline peaks provided evidence of the crystalline nature of the NPs synthesized through a green method.^27^ The average size of the crystallites was calculated with the Scherrer formula.^39^

(3) $$D=\frac{0.9\lambda }{\beta \cos\theta }$$

where D, λ, θ, and β indicate the average crystallite size, X-ray wavelength, Bragg angle, and full width at half maximum. The average crystallite size for α-Fe_2_O_3_ was determined to be 32.03 nm. The Williamson-Hall (W–H) equation was applied to determine the lattice strain (ε)^40^:

(4) βcosθ=KλD+ε(4sinθ)

The strain induced within the crystal from defects in its structure was determined by calculation of the plot slope along β cos θ on the y-axis and 4 sin θ on the x-axis for every peak of the XRD pattern, as shown in Figure 4 (II). The crystallite sizes derived from the Scherrer equation were significantly smaller than those calculated via the W–H equation, indicating a systematic difference attributed to the neglect of microstrain and instrumental broadening contributions in the Scherrer analysis, which are inherently accounted for in the W–H equation. The difference occurred because the W–H equation incorporates microstrain, whereas the Scherrer method assesses the coherence length of X-rays. Consequently, samples with inconsistencies and vacancies display smaller crystallite sizes than their actual dimensions.^40^ The dislocation density was calculated with eq. (5):^39^

(5) $$S=\frac{1}{D^{2}}$$

where S indicates dislocation density, and D indicates the average distance between dislocations. The observed dislocation density was 0.001.

#### Morphological analysis

**Figure 5:**
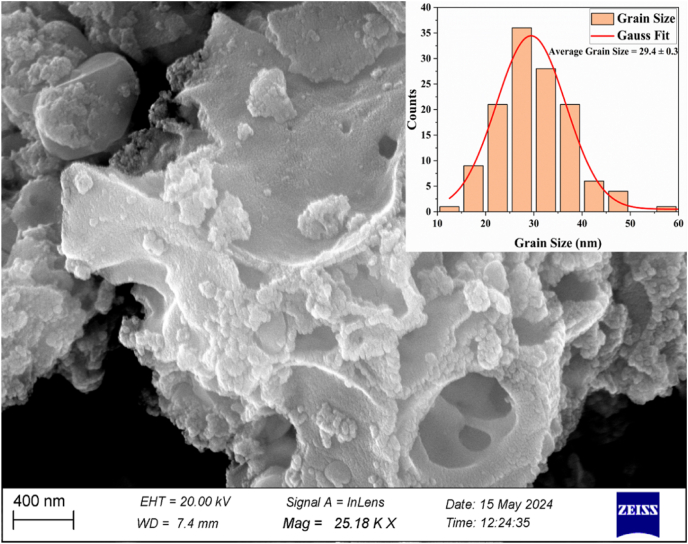
SEM image and average grain size distribution of α-Fe_2_O_3_ NPs.

Figure 5 displays the SEM image and the distribution of grain sizes for the α-Fe_2_O_3_ NPs. Minuscule crystallites spontaneously arranged into larger grains with an uneven form morphology. SEM revealed agglomeration when smaller particles coalesced into larger grains. The mean granule size was 29.4 ± 0.3 nm. These results were consistent with TEM-based findings reported in earlier studies (Supplementary Table S1).

### Antibacterial activity

#### MIC and MBC evaluation

The α-Fe_2_O_3_ NPs exhibited a lower MIC than the rhododendron flower extract (Table 1), thus indicating superior antibacterial action to that of rhododendron flower extract against *B. subtilis*, *S. aureus*, *P. aeruginosa*, and *S. typhi*. Rhododendron extract also exhibited antibacterial activity but was ineffective against *S. typhi*. The augmented activity of NPs might be attributable to their bactericidal interaction, arising from the antimicrobial phenolics and other biochemicals in rhododendron flowers.^41^^,^^42^

**Table 1:** MIC and MBC of rhododendron extract and green synthesized α-Fe_2_O_3_ NPs.

Microbial strains	MIC		MBC	
	Rhododendron extract (mg/mL)	α-Fe_2_O_3_ (mg/mL)	Rhododendron extract (mg/mL)	α-Fe_2_O_3_ (mg/mL)
*Bacillus subtilis*	4	6.25	8	12.5
*Staphylococcus aureus*	8	6.25	16	12.5
*Pseudomonas aeruginosa*	16	12.5	32	25
*Salmonella typhi*	–	12.5	–	25

#### Agar well diffusion method

**Figure 6:**
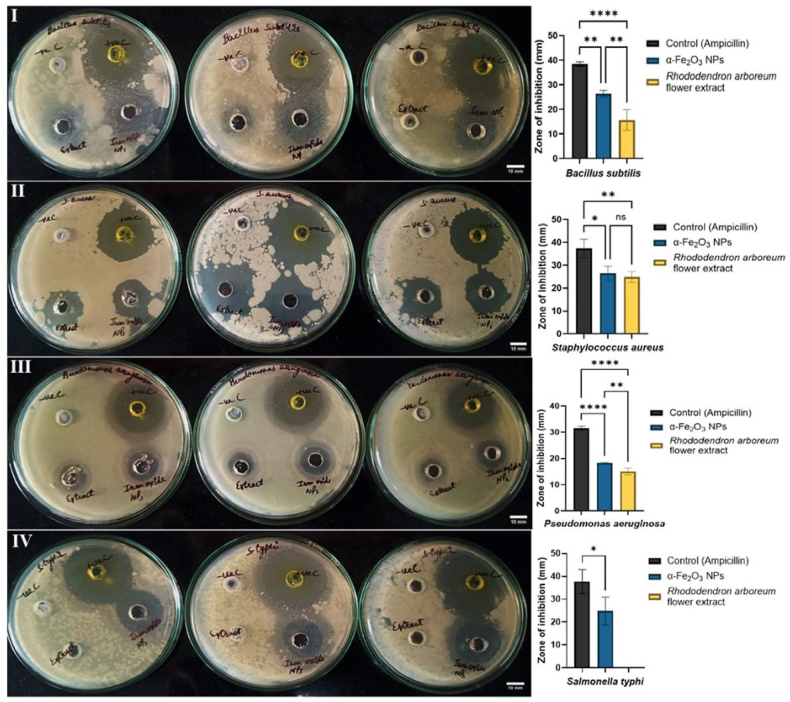
Antibacterial activity and zone of inhibition of α-Fe_2_O_3_ NPs against pathogenic bacteria I) *Bacillus subtilis*, II) *Staphylococcus aureus* (MSSA ATCC 25923), and III) *Pseudomonas aeruginosa* (ATCC 27853), IV) *Salmonella typhi* (ATCC 6539) with *Rhododendron arboreum* flower extract (50 mg/mL), α-Fe_2_O_3_ NPs (30 mg/mL), ampicillin (positive control), and DMSO (negative control). Every value indicates the average ± standard deviation of three replicates. Values are presented as mean ± SD (n = 4). ∗∗∗∗p < 0.0001; ns, non significant p value (negative control).

Subsequently, the α-Fe_2_O_3_ NPs suspensions produced through green methods were evaluated for antibacterial efficacy against *B. subtilis*, *S. aureus*, *P. aeruginosa*, and *S. typhi* through a well-diffusion technique. Figure 6 (I and II) illustrates the efficacy of the α-Fe_2_O_3_ NPs. The produced NPs exhibited distinct antibacterial effects. The largest ZOI was recorded for *S. aureus* (26.55 ± 1.76 mm), whereas the smallest was recorded for *P. aeruginosa* (18.35 ± 0.17 mm). For rhododendron flower extract, the highest ZOI was observed for *S. aureus* (24.8 ± 1.39), whereas the minimum inhibition was observed for *P. aeruginosa* (15.03 ± 1.3 mm). The extract showed no inhibition of *S. typhi.* The green synthesized α-Fe_2_O_3_ NPs showed considerable antibacterial effects with increasing doses. Moreover, the rhododendron extract exhibited antibacterial effects arising from its phytochemical composition.

The antibacterial efficacy of the synthesized α-Fe_2_O_3_ NPs and *Rhododendron arboreum* flower extract against four bacterial strains was determined by measurement of the zones of inhibition (Table 2). The α-Fe_2_O_3_ NPs exhibited substantial antibacterial activity, and the highest ZOI was observed against *S. aureus* and *B*. *subtilis*, followed by *S*. *typhi* and *P. aeruginosa*. In comparison, the flower extract showed moderate inhibition, with ZOIs of 24.8 ± 1.39 mm for *S. aureus*, 15.65 ± 4.17 mm for *B. subtilis*, and 15.03 ± 1.3 mm for *P. aeruginosa*; no inhibition was recorded for *S. typhi*. The negative control (DMSO) showed no antibacterial activity, whereas the positive control demonstrated significantly higher ZOIs across all tested strains. These findings suggested that the α-Fe_2_O_3_ NPs had enhanced antibacterial activity, particularly against Gram-positive bacteria.

**Table 2:** Zone of inhibition of α-Fe_2_O_3_ and *Rhododendron arboreum* flower extract against various bacterial strains (mean ± SD).

Strains	α-Fe_2_O_3_ (ZOI in mm)	Positive control (ZOI in mm)	Negative control (DMSO)	*Rhododendron arboreum* flower extract (ZOI in mm)
*Bacillus subtilis*	26.37 ± 1.31	38.35 ± 0.94	0	15.65 ± 4.17
*Staphylococcus aureus*	26.55 ± 1.76	37.39 ± 4.04	0	24.8 ± 1.39
*Pseudomonas aeruginosa*	18.35 ± 0.17	31.53 ± 0.78	0	15.03 ± 1.3
*Salmonella typhi*	24.85 ± 6.11	37.74 ± 5.26	0	–

**Figure 7:**
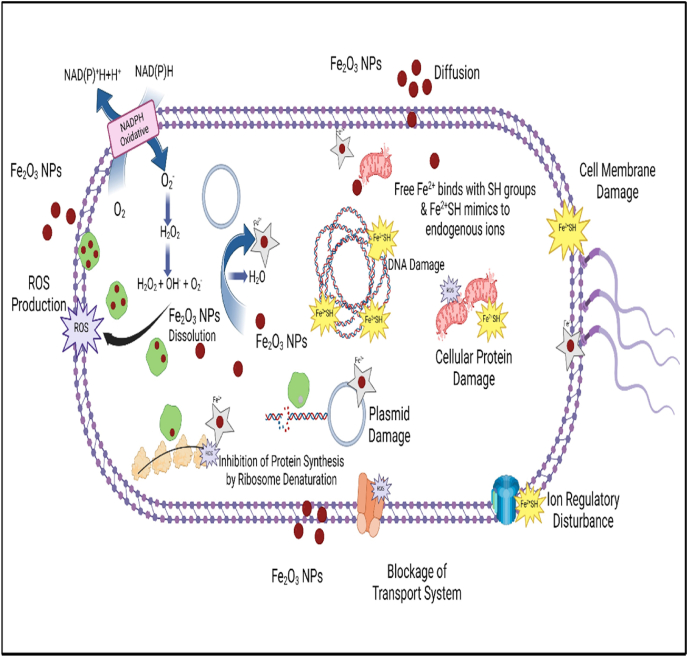
Schematic of antibacterial activity.

The antibacterial activity of α-Fe_2_O_3_ NPs against bacteria involves NP accumulation on the bacterial cell membrane. The formation of electrostatic interactions alters the orientation of cell wall molecules and subsequently disrupts the membrane. Because bacterial membranes contain a lipid layer and lipopolysaccharides, the NPs modify their hydrophobicity, thus causing membrane fluidization and gaining access through membrane pores.^43^ After the NPs enter the cell, they cause leakage of cytoplasmic contents and bind to the amino, carboxyl, and thiol groups of cellular proteins, enzymes, and ion channels. This process induces oxidative stress, structural collapse, and cell death (Figure 7).

#### Anti-inflammatory activity

Protein denaturation occurs predominantly in individuals with arthritic and inflammatory conditions.^44^^,^^45^ Protein denaturation is a well-examined process commonly observed under stress conditions, including exposure to organic solvents, concentrated acids or bases, excessive salt concentrations, or heat.^46^ Protein denaturation may lead to loss of biological activity and structural integrity.^47^ NPs synthesized from plant extracts include phytochemicals with strong anti-inflammatory effects, which are promising candidates for developing pharmaceuticals to treat inflammatory illnesses.^48^

**Figure 8:**
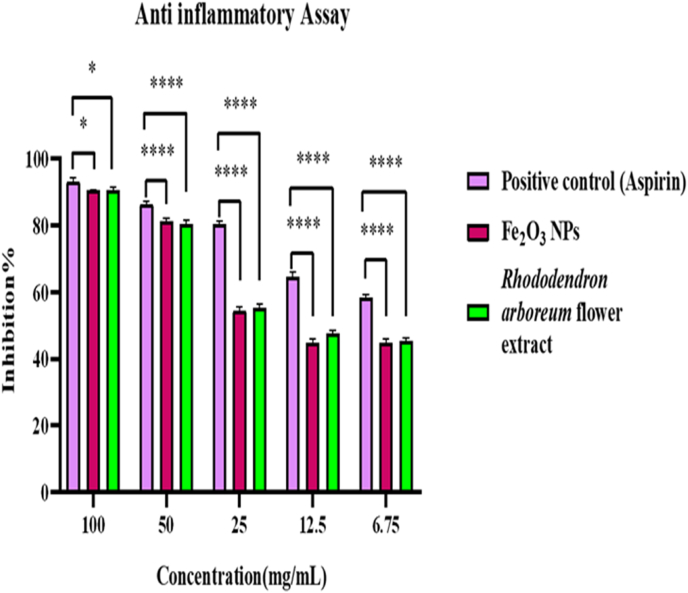
(I) Anti-inflammatory activity of α-Fe_2_O_3_ NPs and plant extract versus positive control. Values are presented as mean ± SD (n = 3). ∗∗∗∗p < 0.0001, ∗∗∗p < 0.001.

We next examined the efficacy of α-Fe_2_O_3_ NPs in preventing the denaturation of egg albumin protein at elevated temperatures across various dosages. The tested NPs demonstrated varying inhibitory activity, depending on the concentration, as shown in Figure 8 (I). The analytical results demonstrated a dose-dependent relationship between the amount of green synthesized NPs and their anti-inflammatory efficacy: the anti-inflammatory activity intensified with increasing concentrations from 6.75 to 100 μg/mL.

#### Assessment of cytotoxicity and anticancer effects of Fe_2_O_3_ NPs in MCF-7 cells

**Figure 9:**
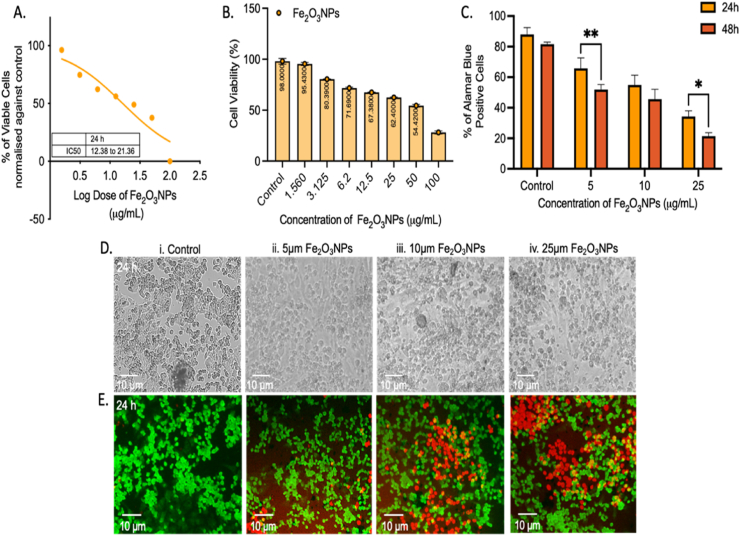
MTT assays depicting the non-linear regression analysis of Fe_2_O_3_ NPs and the IC-50 range (A). (B) Dose-dependent inhibitory effect of Fe_2_O_3_ NPs on MCF-7 cells. (C) Alamar blue assays indicate that Fe_2_O_3_ NP treatments decrease cancer cell proliferation. The morphological assessment is shown in the phase contrast image of Fe_2_O_3_ NP-treated cells (D). AO/PI live staining, showing early apoptotic cells and nuclear damage after Fe_2_O_3_ NP treatments of 0, 5, 10, or 25 μg/mL (E). All images were captured at 100× magnification. (Scale bar, 10 μm).

Cytotoxicity and Alamar blue assays were conducted only on the end product of Fe_2_O_3_ NPs synthesized from plant extract. The cell viability assays of Fe_2_O_3_ NPs at various concentrations in the MCF-7 breast cancer cell line (0–100 μg/mL) confirmed the diminished proliferation trends indicated by the MTT analysis. Non-linear regression analysis was performed on the percentage viable cell data. The 50 % inhibitory concentration (IC-50) of Fe_2_O_3_ NPs was determined to be 22 μg/mL (Figure 9 A and B). Alamar blue assays were conducted to evaluate the potential anticancer effects of Fe_2_O_3_ NPs in MCF-7 cells after 24- and 48-h Fe_2_O_3_ NP (1–25 μg/mL) treatments. Concentrations of 5 μg/mL and 25 μg/mL Fe_2_O_3_NP significantly inhibited the proliferation of cancer cells at 48 h versus 24 h (Figure 9 C). This finding suggests that Fe_2_O_3_ NPs are a promising anticancer material for breast cancer treatment.

In addition, AO/PI staining illustrated the presence of live (green) and dead (red) cancer cells in the Fe_2_O_3_ NP treatments, thus demonstrating that induction with Fe_2_O_3_ NPs notably initiated cell death mechanisms in the cancer cells (Figure 9D and E). This analysis indicated that Fe_2_O_3_ NPs significantly inhibited cancer cell proliferation and confirmed their anticancer properties against the MCF-7 cell line.

## Discussion

The use of *Rhododendron arboreum* flower extract for the green synthesis of α-Fe_2_O_3_ NPs is a novel approach not previously reported in the literature. This plant is known for its rich phytochemical content, including flavonoids, phenolics, and other antioxidant compounds, which play crucial roles as natural reducing and stabilizing agents in NP synthesis.^49^^,^^50^ The distinctive composition of this floral extract might have contributed to the observed morphology and surface charge characteristics of the synthesized NPs, which in turn influence their biological activity.^51^ By introducing a new plant-based route for NP synthesis, this study adds to the growing body of green nanotechnology literature by using an ecologically friendly and underexplored botanical resource.^52^

A key strength of this study is its thorough physicochemical characterization of the synthesized α-Fe_2_O_3_ NPs. Multiple analytical techniques, including XRD, SEM, EDX, UV-visible spectroscopy, FTIR, DLS, and zeta potential analysis, were used to confirm the crystalline structure, elemental composition, morphology, particle size distribution, and surface charge. The average crystallite size of 32.03 nm, combined with a hydrodynamic diameter of 274.7 nm and a zeta potential of −18.6 mV, indicated stable colloidal properties and nanoscale dimensions suitable for biomedical applications.^53^ This multi-technique approach provided a comprehensive profile of the NPs, including crucial insights into their structural integrity and biological behaviour—an aspect often underexplored in similar green synthesis studies.

Beyond synthesis and characterization, the current study evaluated the multifunctional bioactivity of the α-Fe_2_O_3_ NPs. The NPs exhibited potent antibacterial effects against both Gram-positive and Gram-negative pathogens, significant anti-inflammatory activity, as demonstrated by protein denaturation inhibition, and strong cytotoxic potential against MCF-7 breast cancer cell lines, with an IC_50_ value of 22 μg/mL.^54^, ^55^, ^56^, ^57^ This broad-spectrum activity highlights the biomedical relevance of the synthesized NPs and positions them as promising candidates for multifunctional therapeutic applications.^58^, ^59^, ^60^ Such a comprehensive biological evaluation is relatively rare in similar studies, thereby enhancing the translational value and novelty of the current work.

The synthesized α-Fe_2_O_3_ NPs demonstrated significantly greater antibacterial activity than the crude *Rhododendron arboreum* flower extract, as reflected by the lower MIC and MBC values across tested bacterial strains. This increase in efficacy was attributed to the nanoscale size, increased surface area, and possible synergistic effects arising from bioactive phytochemicals capping the NPs.^61^^,^^62^ This enhancement suggested that the synthesis process not only conserved the therapeutic properties of the plant extract but also potentially amplified them through NP formulation. This finding supports the potential of green-synthesized NPs as improved alternatives to crude plant extracts for biomedical applications, including antimicrobial therapy.

Despite the successful green synthesis of multifunctional α-Fe_2_O_3_ NPs with *Rhododendron arboreum* flower extract, several limitations must be acknowledged. Notably, long-term NP stability was not assessed beyond the immediate post-synthesis phase. The lack of time-course data on zeta potential and aggregation behavior limits insights into their shelf life and dispersion stability, which are crucial for biomedical applications. Additionally, although the synthesized NPs demonstrated notable antibacterial activity, mechanistic analyses, such as reactive oxygen species generation or bacterial membrane integrity assays, were not performed. Incorporating these studies into future work would help elucidate the precise antimicrobial mechanisms. Similarly, although cytotoxic effects on MCF-7 breast cancer cells were confirmed through MTT and Alamar blue assays, we did not explore apoptosis-related pathways with tools such as flow cytometry or western blotting. The literature has suggested a caspase-3/Bax-mediated apoptosis for similar green synthesized Fe_2_O_3_ NPs (Supplementary Table S2); however, dedicated experiments are needed to validate this mechanism for *Rhododendron arboreum*-derived NPs. The anti-inflammatory activity, assessed via egg albumin denaturation assays, provided preliminary evidence but did not capture the complexity of cellular immune responses. More sophisticated assays, such as nitric oxide inhibition and cytokine profiling in macrophages, would offer deeper insights. Although the NPs did not exhibit significantly greater anti-inflammatory activity than the plant extract, their improved stability, biocompatibility, and potential for targeted delivery highlight the need for further investigation. Future studies should focus on optimizing formulations and validating the findings in physiologically relevant models to better harness the biomedical potential of these green-synthesized NPs.

## Conclusion

In this study, we successfully synthesized α-Fe_2_O_3_ NPs by using *Rhododendron arboreum* flower extract, through an ecologically friendly, green synthesis approach. The NPs were thoroughly characterized, and demonstrated promising antibacterial, anti-inflammatory, and anticancer activities. Notably, the antibacterial activity was most pronounced against *B. subtilis*, whereas the effects against the other tested bacterial strains were comparatively moderate. This finding highlights a need for broader-spectrum antibacterial evaluations involving a wider range of pathogens and additional mechanistic assays. Although some biological responses, such as anti-inflammatory effects, did not show significant enhancement with respect to the plant extract, the NPs possess favourable characteristics, including enhanced stability, biocompatibility, and cytotoxicity against MCF-7 cells. These attributes highlight their potential for biomedical applications. Future studies should focus on expanding antimicrobial assessments, validating cellular and molecular mechanisms (e.g., apoptosis and inflammation), and exploring in vivo models to better understand the therapeutic potential of *Rhododendron arboreum*-mediated iron oxide NPs.

## Ethical approval

Not applicable.

## Authors contributions

Conceptualisation, KMB, IS; Methods, IS, VKD, and AC; Sample analysis, IS and AC; Data curation, SK and RS; Writing—original draft preparation, IS; Writing—review and editing, AC, VKD, RV, KMB, and SH; Supervision, SH and KMB; Project administration, AC; Funding acquisition, KMB and AAI. All authors have critically reviewed and approved the final draft and are responsible for the content and similarity index of the manuscript.

## Source of funding

This work was supported by the Researchers Supporting Project (number RSPDR993).

## Conflict of interest

The authors declare no conflicts of interest.
