# Supplementary material for: From flowers to function: Structural and biomedical exploration of iron oxide nanoparticles synthesized from Rhododendron arboreum extract
Source: J Taibah Univ Med Sci. 2025 Oct 16;20(5):737–50. doi: 10.1016/j.jtumed.2025.09.005 (PMC12552967; doi:10.1016/j.jtumed.2025.09.005)
Supplement: Multimedia component 1 [file mmc1.docx]

**Supplementary table S1: TEM-based findings for plant-synthesized Fe₂O₃ NPs:**

| **Plant** | **Part used** | **Size and morphology** | **Reference** |
| --- | --- | --- | --- |
| Camellia sinensis | Leaf | 5–15 nm Spherical crystalline | Hoag, G.E.; Collins, J.B.; Holcomb, J.L.; Hoag, J.R.; Nadagouda, M.N.; Varma, R.S. Degradation of bromothymol blue by ‘greener’ nano-scale zero-valent iron synthesized using tea polyphenols. *J. Mater. Chem.* **2009**, *19*, 8671–8677. [[**Google Scholar**](http://scholar.google.com/scholar_lookup?title=Degradation+of+bromothymol+blue+by+%E2%80%98greener%E2%80%99+nano-scale+zero-valent+iron+synthesized+using+tea+polyphenols&author=Hoag,+G.E.&author=Collins,+J.B.&author=Holcomb,+J.L.&author=Hoag,+J.R.&author=Nadagouda,+M.N.&author=Varma,+R.S.&publication_year=2009&journal=J.+Mater.+Chem.&volume=19&pages=8671%E2%80%938677&doi=10.1039/b909148c)] [**[CrossRef](http://dx.doi.org/10.1039/b909148c)**] |
| Green tea | Leaf | 40–60 nm amorphous | Shahwan, T.; Abu Sirriah, S.; Nairat, M.; Boyacı, E.; Eroğlu, A.E.; Scott, T.B.; Hallam, K.R. Green synthesis of iron nanoparticles and their application as a fenton-like catalyst for the degradation of aqueous cationic and anionic dyes. *Chem. Eng. J.* **2011**, *172*, 258–266. [[**Google Scholar**](http://scholar.google.com/scholar_lookup?title=Green+synthesis+of+iron+nanoparticles+and+their+application+as+a+fenton-like+catalyst+for+the+degradation+of+aqueous+cationic+and+anionic+dyes&author=Shahwan,+T.&author=Abu+Sirriah,+S.&author=Nairat,+M.&author=Boyac%C4%B1,+E.&author=Ero%C4%9Flu,+A.E.&author=Scott,+T.B.&author=Hallam,+K.R.&publication_year=2011&journal=Chem.+Eng.+J.&volume=172&pages=258%E2%80%93266&doi=10.1016/j.cej.2011.05.103)] [**[CrossRef](http://dx.doi.org/10.1016/j.cej.2011.05.103)**] |
| Eucalyptus Tereticornis | Leaf | 40–60 nm Cubic | Wang, Z. Iron complex nanoparticles synthesized by eucalyptus leaves. *ACS Sustain. Chem. Eng.* **2013**, *1*, 1551–1554. [[**Google Scholar**](http://scholar.google.com/scholar_lookup?title=Iron+complex+nanoparticles+synthesized+by+eucalyptus+leaves&author=Wang,+Z.&publication_year=2013&journal=ACS+Sustain.+Chem.+Eng.&volume=1&pages=1551%E2%80%931554&doi=10.1021/sc400174a)] [**[CrossRef](http://dx.doi.org/10.1021/sc400174a)**] |
| Hordeum vulgare and Rumex acetosa | Leaf | 10–40 nm amorphous | Makarov, V.V.; Makarova, S.S.; Love, A.J.; Sinitsyna, O.V.; Dudnik, A.O.; Yaminsky, I.V.; Taliansky, M.E.; Kalinina, N.O. Biosynthesis of stable iron oxide nanoparticles in aqueous extracts of *Hordeum vulgare* and *Rumex acetosa* plants. *Langmuir* **2014**, *30*, 5982–5988. [[**Google Scholar**](http://scholar.google.com/scholar_lookup?title=Biosynthesis+of+stable+iron+oxide+nanoparticles+in+aqueous+extracts+of+Hordeum+vulgare+and+Rumex+acetosa+plants&author=Makarov,+V.V.&author=Makarova,+S.S.&author=Love,+A.J.&author=Sinitsyna,+O.V.&author=Dudnik,+A.O.&author=Yaminsky,+I.V.&author=Taliansky,+M.E.&author=Kalinina,+N.O.&publication_year=2014&journal=Langmuir&volume=30&pages=5982%E2%80%935988&doi=10.1021/la5011924&pmid=24784347)] [**[CrossRef](http://dx.doi.org/10.1021/la5011924)**] [[**PubMed**](http://www.ncbi.nlm.nih.gov/pubmed/24784347)] |
| GarlicVine (Mansoa alliacea) | Leaf | 13.82 nm–15.45 nm crystalline | Prasad, A.S. Iron oxide nanoparticles synthesized by controlled bio-precipitation using leaf extract of garlic vine (*Mansoa alliacea*). *Mater. Sci. Semicond. Process.* **2016**, *53*, 79–83. [[**Google Scholar**](http://scholar.google.com/scholar_lookup?title=Iron+oxide+nanoparticles+synthesized+by+controlled+bio-precipitation+using+leaf+extract+of+garlic+vine+(Mansoa+alliacea)&author=Prasad,+A.S.&publication_year=2016&journal=Mater.+Sci.+Semicond.+Process.&volume=53&pages=79%E2%80%9383&doi=10.1016/j.mssp.2016.06.009)] [**[CrossRef](http://dx.doi.org/10.1016/j.mssp.2016.06.009)**] |
| Passiflora tripartitavar | Fruit | 18.23–24.65 nm spherical crystalline | Kumar, B.; Smita, K.; Cumbal, L.; Debut, A. Biogenic synthesis of iron oxide nanoparticles for 2-arylbenzimidazole fabrication. *J. Saudi Chem. Soc.* **2014**, *18*, 364–369. [[**Google Scholar**](http://scholar.google.com/scholar_lookup?title=Biogenic+synthesis+of+iron+oxide+nanoparticles+for+2-arylbenzimidazole+fabrication&author=Kumar,+B.&author=Smita,+K.&author=Cumbal,+L.&author=Debut,+A.&publication_year=2014&journal=J.+Saudi+Chem.+Soc.&volume=18&pages=364%E2%80%93369&doi=10.1016/j.jscs.2014.01.003)] [**[CrossRef](http://dx.doi.org/10.1016/j.jscs.2014.01.003)**] |
| Syzygium cumini | Seed | 9–20 nm spherical crystalline | Venkateswarlu, S.; Natesh Kumar, B.; Prasad, C.H.; Venkateswarlu, P.; Jyothi, N.V.V. Bio-inspired green synthesis of Fe_3_O_4_ spherical magnetic nanoparticles using *Syzygium cumini* seed extract. *Physica. B* **2014**, *449*, 67–71. [[**Google Scholar**](http://scholar.google.com/scholar_lookup?title=Bio-inspired+green+synthesis+of+Fe3O4+spherical+magnetic+nanoparticles+using+Syzygium+cumini+seed+extract&author=Venkateswarlu,+S.&author=Natesh+Kumar,+B.&author=Prasad,+C.H.&author=Venkateswarlu,+P.&author=Jyothi,+N.V.V.&publication_year=2014&journal=Physica.+B&volume=449&pages=67%E2%80%9371&doi=10.1016/j.physb.2014.04.031)] [**[CrossRef](http://dx.doi.org/10.1016/j.physb.2014.04.031)**] |
| Salvia officinalis | Leaf | 5–25 nm spherical | Wang, Z.; Fang, C.; Mallavarapu, M. Characterization of iron–polyphenol complex nanoparticles synthesized by sage (*Salvia officinalis*) leaves. *Environ. Technol. Innov.* **2015**, *4*, 92–97. [[**Google Scholar**](http://scholar.google.com/scholar_lookup?title=Characterization+of+iron%E2%80%93polyphenol+complex+nanoparticles+synthesized+by+sage+(Salvia+officinalis)+leaves&author=Wang,+Z.&author=Fang,+C.&author=Mallavarapu,+M.&publication_year=2015&journal=Environ.+Technol.+Innov.&volume=4&pages=92%E2%80%9397&doi=10.1016/j.eti.2015.05.004)] [**[CrossRef](http://dx.doi.org/10.1016/j.eti.2015.05.004)**] |
| Dodonaea viscose | Leaf | 50–60 nm Spherical | Kiruba Daniel, S.C.G.; Vinothini, G.; Subramanian, N.; Nehru, K.; Sivakumar, M. Biosynthesis of Cu, ZVI, and Ag nanoparticles using *Dodonaea viscosa* extract for antibacterial activity against human pathogens. *J. Nanopart. Res.* **2012**, *15*, 1319. [[**Google Scholar**](http://scholar.google.com/scholar_lookup?title=Biosynthesis+of+Cu,+ZVI,+and+Ag+nanoparticles+using+Dodonaea+viscosa+extract+for+antibacterial+activity+against+human+pathogens&author=Kiruba+Daniel,+S.C.G.&author=Vinothini,+G.&author=Subramanian,+N.&author=Nehru,+K.&author=Sivakumar,+M.&publication_year=2012&journal=J.+Nanopart.+Res.&volume=15&pages=1319&doi=10.1007/s11051-012-1319-1)] [**[CrossRef](http://dx.doi.org/10.1007/s11051-012-1319-1)**] |

**Supplementary table S2: Comparative Analysis of Apoptotic Markers Induced by Green-Synthesized Fe₂O₃ NPs**

| **Plant Extract Used** | **Cell Line Tested** | **Key Apoptotic Markers** | **Observed Effects** | **Reference** |
| --- | --- | --- | --- | --- |
| *Moringa oleifera* | MCF-7 (Breast cancer) | ↑ Caspase-3, ↑ Bax, ↓ Bcl-2 | 2.5-fold caspase-3 activation at 50 µg/mL; Bax/Bcl-2 ratio increased by 3x | doi: 10.1016/j.jphotobiol.2020.111940. Epub 2020 Jun 21. PMID: 32603875. |
| *Azadirachta indica* (Neem) | A549 (Lung cancer) | ↑ Cleaved caspase-3, ↑ Bax/Bcl-2 ratio | Dose-dependent caspase-3 cleavage (80% at 100 µg/mL) | Subapriya R, Bhuvaneswari V, Nagini S. Ethanolic neem (Azadirachta indica) leaf extract induces apoptosis in the hamster buccal pouch carcinogenesis model by modulation of Bcl-2, Bim, caspase 8 and caspase 3. Asian Pac J Cancer Prev. 2005 Oct-Dec;6(4):515-20. PMID: 16436003. |
| *Ocimum sanctum* (Tulsi) | HeLa (Cervical cancer) | ↑ Caspase-9, ↑ Bax, ↓ Bcl-2 | Mitochondrial apoptosis pathway confirmed via WB | Akhter KF, Mumin MA, Lui EMK, Charpentier PA. Transdermal nanotherapeutics: Panax quinquefolium polysaccharide nanoparticles attenuate UVB-induced skin cancer. Int J Biol Macromol. 2021 Jun 30;181:221-231. doi: 10.1016/j.ijbiomac.2021.03.122. Epub 2021 Mar 24. PMID: 33774070. |
| *Eucalyptus globulus* | HT-29 (Colon cancer) | ↑ Caspase-3/7 activity, ↑ ROS | 4-fold higher apoptosis vs. control (flow cytometry) | Zhang L, Li C, Peng D, Yi X, He S, Liu F, Zheng X, Huang WE, Zhao L, Huang X. Raman spectroscopy and machine learning for the classification of breast cancers. Spectrochim Acta A Mol Biomol Spectrosc. 2022 Jan 5;264:120300. doi: 10.1016/j.saa.2021.120300. Epub 2021 Aug 21. PMID: 34455388. |
| *Green tea* (Camellia sinensis) | PC-3 (Prostate cancer) | ↑ Bax, ↓ Bcl-2, PARP cleavage | Synergistic effect with phytochemicals | Zhe Cheng, Zhifa Zhang, Yu Han, Jing Wang, Yongyong Wang, Xiaoqiang Chen, Yundong Shao, Yong Cheng, Weilong Zhou, Xiaolei Lu, Zhengqi Wu, |
| Current Study (*Rhododendron arboreum*) | [MCF-7 cells] | *Predicted*: ↑ Bax/Bcl-2, caspase-3 activation | *Inferred from viability assays* | – |
